# Corneal Cross-linking for Keratoconus: Exploring the Issues Regarding Accelerated Protocols and Thin Corneas

**DOI:** 10.18502/jovr.v16i3.9425

**Published:** 2021-07-29

**Authors:** Farhad Hafezi

**Affiliations:** ^1^Ocular Cell Biology Group, Center for Applied Biotechnology and Molecular Medicine, University of Zurich, Switzerland; ^2^ELZA Institute AG, Dietikon/Zurich, Switzerland; ^3^Department of Ophthalmology, University of Southern California, Los Angeles, CA, USA; ^4^Faculty of Medicine, University of Geneva, Geneva, Switzerland; ^5^Department of Ophthalmology, Wenzhou Medical University, Wenzhou, China

The current issue of *Journal of Ophthalmic and Vision Research* (*JOVR*) observes the publication of two useful additions to the cross-linking literature: an *in vitro* confocal microscopy (IVCM) evaluation of structural changes that occur in thin keratoconic corneas following corneal cross-linking (CXL),^[1]^ and an evaluation of accelerated versus standard Dresden protocol CXL for treatment of progressive keratoconus in Syria.^[2]^

It is worth briefly reviewing the history of CXL. For over 20 years, CXL has been used for the treatment of corneal ectatic disorders such as keratoconus, and involves saturating the corneal stroma with riboflavin, followed by a period of ultraviolet (UV)-A irradiation to photoactivate the riboflavin and cause it to covalently cross-link molecules within the corneal stroma (principally collagen, but also other molecules such as glycoproteins) and strengthen the cornea with the intention of halting the progression of ectasia.^[3]^ Epithelial cells at the surface of the cornea are effective barriers against riboflavin penetration into the stroma, therefore these cells are typically debrided before riboflavin is applied (the “epi-off” approach). The original CXL protocol, developed 20 years ago, called the Dresden protocol, is an epi-off protocol that involves irradiation of a riboflavin-saturated cornea with 30 min of UV-A light at an intensity of 3 mW/cm².^[4]^ This delivers a total UV-A fluence (or “dose”) of 5.4 J/cm² and is still considered to be the gold standard for strengthening ectatic corneas.

Nevertheless, the Dresden protocol has a number of drawbacks. For safety reasons and to protect corneal endothelial cells from potentially dangerous levels of UV-A exposure, only corneas 400 µm or thicker may be treated with the Dresden protocol.^[4]^ In addition, 30 min of UV-A irradiation is a long period and therefore accelerated protocols that increase the intensity of illumination with a proportionate reduction in irradiation time have been pursued. However, the discovery that oxygen is an essential component of the cross-linking phenomenon (and its diffusion into the stroma is a rate-limiting step) explains why increased intensity-reduced time irradiation protocols result in less effective cross-linking.^[5,6,7,8]^ The current consensus is that the most reasonable trade-off lies around 10 min of 9 mW/cm² irradiation which still provides most of the effect of the full 30 min irradiation protocol while cutting treatment time to one-third.^[8]^

The article by Salman et al^[2]^ published in this issue of *JOVR* reports a trial that compared an accelerated epi-off CXL irradiation protocol (10 mW/cm² for 9 min) with the standard Dresden protocol (3 mW/cm² for 30 min). Amongst the visual, corneal tomographic and topographic factors examined, the researchers noted that “this [was] the first study to compare the impact of different CXL protocols on posterior corneal astigmatism.” They found that accelerated CXL resulted in significantly greater anterior corneal flattening, a greater increase in posterior steepening, a larger decrease in posterior astigmatism, and a greater reduction in minimum thickness than the standard CXL protocol. They noted that both protocols showed improvement in postoperative visual acuity (VA) and higher order aberrations, but the accelerated CXL protocol resulted in significantly better VA than the standard protocol.

Although this study was performed on a relatively small sample (63 eyes, 40 patients), it adds to the body of evidence that accelerated CXL protocols might result in a greater flattening effect than standard CXL protocols, which is an encouraging finding indicating that accelerated CXL protocols might offer visual rehabilitation in subjects with ectatic corneas, as flattening is associated with a reduction in ectasia-associated myopia.

Corneal ectasias, however, can result in corneas that are thinner than 400 µm, and if these cases cannot be treated with CXL, they may ultimately require keratoplasty. A number of approaches have been taken to artificially thicken the cornea,^[9]^ either through swelling the cornea to a thickness ≥ 400 µm using hypotonic riboflavin prior to and during UV-A irradiation^[10]^ or through the placement of a riboflavin-soaked contact lens to artificially bulk the cornea.^[11]^ Both approaches have drawbacks, that is, less predictable or suboptimal strengthening outcomes,^[5]^ but can still offer corneal strengthening and some level of protection against later ectasia progression.

The IVCM paper by Sufi et al^[1]^ offers some insight on the use of hypertonic riboflavin to cross-link thin corneas. In this paper, 10 eyes from 10 patients with progressive keratoconus and corneal thickness ranging from 350 to 399 µm received CXL with hypotonic riboflavin and were examined by IVCM at postoperative months 1, 3, and 6. The authors observed evidence of edema at one month, which reduced gradually over the next five months; that the subepithelial nerve plexus was completely absent one month after CXL, but regenerated gradually over the next five months (albeit poorly in 2 out of 10 eyes); and stromal keratocyte apoptosis in the anterior stroma in all cases (which extended to the posterior stroma in four eyes) with gradual repopulation over the remainder of the study course. However, it was clear that thin cornea CXL significantly decreased the specular endothelial cell count by 8%, albeit without signs of endothelial trauma.

The study by Sufi et al^[1]^ is a small report with its inherent limitations, but raises important questions about the UV dose applied to thin ( < 400 µm) corneas during cross-linking: there is less tissue in these thin corneas, the increased mass is simply 0.1% riboflavin solution. Although riboflavin absorbs UV energy (and is consumed during the process), it may be the case that the tissue bulk in riboflavin-soaked corneas naturally thicker than 400 µm absorbs more UV energy and therefore shields the endothelial cells from UV-A energy better than the relatively sparse tissue in thin corneas artificially thickened by hypotonic riboflavin solution. Fortunately, a newer approach is now available that adjusts the total fluence of UV-A based on immediate pre-irradiation corneal thickness; the so-called sub400 protocol^[12]^ delivers a UV dose intended to avoid irradiating the 70 µm posterior stroma safety zone first defined in the Dresden protocol without resorting to artificial thickening approaches. The sub400 protocol can even be used with the simplest first-generation cross-linking machines by only altering the duration of UV-A irradiation.
